# Outcomes of neoadjuvant chemoradiotherapy in Japanese locally advanced rectal carcinoma patients

**DOI:** 10.1186/s12957-016-0898-1

**Published:** 2016-04-30

**Authors:** Katsuji Tokuhara, Yosuke Ueyama, Kazuyoshi Nakatani, Kazuhiko Yoshioka, Masanori Kon

**Affiliations:** Department of Surgery, Kansai Medical University, 10-15 Fumizonocho, Moriguchi, Osaka 570-8507 Japan

**Keywords:** Neoadjuvant chemoradiotherapy, Rectal cancer

## Abstract

**Background:**

We investigated the efficacy and prognosis of neoadjuvant chemoradiotherapy (NACRT) for Japanese locally advanced rectal carcinoma patients.

**Methods:**

Fifty-seven patients diagnosed with cT3-4 or any cT/cN+ disease using enhanced computed tomography or magnetic resonance imaging from 2002 to 2014 were enrolled. The male/female ratio was 42/15, and the median age was 67 years. Ra/Rb/Rb-P/P was expressed by 6/35/14/2 patients. Histological tumor types were tub1/tub2/por/muc in 22/30/4/1 patients. For NACRT, radiotherapy doses were 40–50.4 Gy chemotherapy consisted of 5′-DFUR, capecitabine, or S1.

**Results:**

All 57 patients received curative surgical treatment. The anal preservation rate was 65.0 %. The ypStage of 0/I/II/IIIa/IIIb was 7/10/25/11/4 cases. The histological antitumor effect (HATE) was ≥grade (G) 2 and G3 in 31 (54.4 %) and 7 (12.3 %) cases, respectively. Postoperative complications occurred in 17 patients and exceeded GIII (Clavien–Dindo classification) in four patients. Recurrence was observed in 19 patients; the primary local recurrence rate was 5.3 %. The 3-year relapse-free survival (RFS) and overall survival (OS) rates were 64.8 and 95.5 %, respectively; the 5-year RFS and OS rates were 60.2 and 61.0 %, respectively. In multivariate analysis, ypN+ was a high-risk factor for distant organ recurrence. As predictive factors regarding the efficacy of NACRT, a neutrophil concentration <70 % and a neutrophil/lymphocyte ratio <3.0 in peripheral blood prior to treatment indicated that NACRT would be significantly more effective.

**Conclusions:**

NACRT was effective in reducing local recurrence but did not suppress distant organ recurrence in Japanese locally advanced rectal carcinoma patients. A further investigation of an extension of the NACRT regimen is required.

## Background

The incidence of colorectal cancer has gradually increased. In Japan, this cancer was ranked as the second and fourth most common type among women and men, respectively, in 2014 [1]. Locally advanced rectal cancer (LARC) has often developed postoperative local recurrence. To manage postoperative pelvic local control and downstage LARC, multimodal therapy such as neoadjuvant chemoradiotherapy (NACRT) is frequently used in Western countries [2–4]. Several randomized studies have shown that NACRT provided better local control and was more effective than postoperative radiotherapy [4, 5]. Based on these studies, NACRT for rectal cancer has been recommended for LARC in the NCCN version 2.2015 guidelines [6].

In Japan, the standard treatment for LARC is total mesorectal excision and lateral pelvic lymph node dissection; postoperative outcomes using surgery alone were better than those of Western countries, and consequently, NACRT has not been introduced aggressively. However, in some institutes, adjuvant therapy for LARC such as chemoradiotherapy (CRT) or chemotherapy has been introduced. Several phase II studies regarding the safety and efficacy of NACRT in Japanese patients have been reported [7–9].

In the present study, we investigated the usefulness, efficacy, and prognosis of NACRT in Japanese LARC patients, concerning the histological antitumor effect (HATE) and long-term outcomes such as relapse-free survival (RFS), overall survival (OS), and recurrence rate.

## Methods

Between September 2002 and April 2014, a total of 57 patients with LARC underwent NACRT and total mesorectal excision (TME) at the Department of Surgery in Kansai Medical University Hospital. Inclusion criteria for this retrospective study were LARC patients with cT3-4 or any cT/cN+ disease assessed using colonoscopy, enhanced computed tomography, and magnetic resonance imaging.

NACRT was administered by a multidisciplinary team of radiotherapists and colorectal surgeons. The patients received long-course radiotherapy at two different doses. At the initial stage of the study, the radiation dose was 40 Gy (4 weeks), and at the latter stage, it was 50.4 Gy (5.5 weeks). Radiotherapy was performed using the classic four-field technique. In combination chemotherapy, three different protocols were used. All patients took oral fluorouracil, 5′-DFUR, capecitabine, or S1 only on the day of irradiation.

Surgery was undertaken at 4–6 weeks after NACRT. We performed abdominoperitoneal resection (APR), low anterior resection (LAR), internal sphincteric resection (ISR), and total pelvic floor exenteration (TPE) with TME; surgery was performed with or without lateral pelvic lymph node dissection. All patients provided informed consent. The type of surgery was determined at a preoperative doctor’s conference based on the oncological condition of each patient.

Staging and HATE was performed according to the *Japanese Society for Cancer of the Colon and Rectum* (Eighth Edition) guidelines [10]. The histological tumor response to NACRT was determined using scale grade (G) 0, 1a, 1b, 2, and 3 as follows: HATE G0, no response to treatment; HATE G1a, tumor size reduction of 1/3; HATE G1b, tumor size reduction of 1/3–2/3; HATE G2, tumor size reduction of >2/3; and HATE G3, complete tumor ablation, equal to a pathological complete response (pCR).

To investigate the factors associated with postoperative distant organ recurrence, we analyzed age, sex, type of surgical procedure, histological depth of tumor, HATE grade, radiation dose, type of combination chemotherapy, and presence or absence of pathological lymph node metastasis. To evaluate the predictive factors regarding the efficacy of NACRT, we analyzed the number of leukocytes, the percentage of neutrophils and lymphocytes, the neutrophil-to-lymphocyte ratio (NLR), hemoglobin and albumin levels, platelet count, and carcinoembryonic antigen (CEA) in peripheral blood prior to treatment.

JMP ver.9 software was used for statistical analysis. Continuous variables are presented as the median and range. The univariate analysis data were assessed using the Yates chi-square test. The Cox regression model (logistic regression analysis) was used for multivariate analysis. RFS and OS were analyzed using the log-rank test and were plotted as Kaplan–Meier curves. In all analyses, *p* < 0.05 indicated statistical significance.

## Results

Fifty-seven LARC patients who had no distant metastasis and had undergone histopathologically complete resection (September 2002 to April 2014) with TME were enrolled in the study. Patient demographics, preoperative variables, surgical procedure, and postoperative complications are summarized in Table 1. The study group consisted of 42 men and 15 women with a median age of 67 (range, 40–87) years. The location of the tumor was as follows: 6 patients, Ra; 35 patients, Rb; 14 patients, Rb-P; and 2 patients, P. The median distance from the lower edge of the tumor to the anal verge was 4.0 cm (0–15 cm). Histological tumor types were as follows: 22 patients, well-differentiated tubular adenocarcinoma; 30 patients, moderately differentiated tubular adenocarcinoma; 4 patients, poorly differentiated adenocarcinoma; and 1 patient, mucinous adenocarcinoma. There were 17 patients with clinical stage II, 28 patients with clinical stage IIIa, and 12 patients with clinical stage IIIb disease. Forty patients (70.2 %) had clinical stage III disease. Surgical treatments were as follows: 17 patients, APR (including 5 treated with laparoscopy-assisted surgery (LS)); 10 patients, ISR (including 4 treated with LS); 27 patients, LAR (including 16 treated with LS); and 3 patients, TPE. Although 20 patients underwent bilateral lateral pelvic lymph node dissection, there were no patients with lateral pelvic lymph node metastasis. There were 37 (65.0 %) patients where anal preservation was achieved. Postoperative complications occurred in 17 (29.8 %) patients, but there were only 4 (7.0 %) patients with >grade III (Clavien–Dindo classification) complications.

**Table 1:** Demographics, preoperative variables, surgical procedure, and postoperative complications

Gender (male/female)		42/15
Age (year)		67 (range 40–87)
ASA score	1	53
	2	4
Tumor location	Rb	35
	Rb-P	14
	Ra	6
	P	2
Median distant from lower edge of tumor to anal verge (cm)		4.0 (0~15)
Histological type	tub1	22
	tub2	30
	por	4
	muc	1
Clinical stage	II	17
	IIIa	28
	IIIb	12
Operative procedure (laparoscopic surgery)	Total perineal excision	3
	Abddominoperitoneal resection	17 (5)
	Low anterior resection	27 (16)
	Inferior sphincter resection	10 (4)
No. of anus preserving cases (%)		37 (65.0)
No. of patient with complication (Clavien–Dindo classification score ≥3)	Overall	17 (4)
	Perianal wound infection	2
	Bowel obstruction	4 (1)
	Stoma related complication	3 (1)
	Anastomotic leakage	2 (1)
	Abscess in pelvic cavity	2 (1)
	Chylous ascites	1
	Neurogenic bladder	1
	Sepsis (enterocolitis induced)	1
	Radiational enteritis	1

*ASA* American Society of Anesthesiologists, *tub1* well differentiated tubular adenocarcinoma, *tub2* moderately differentiated tubular adenocarcinoma, *por* poorly differentiated adenocarcinoma, *muc* mucinous adenocarcinoma

Details of the NACRT regimen, its therapeutic effects, and cases of recurrence after surgery are summarized in Table 2. As part of the NACRT regimen, radiotherapy was delivered in four fields at a total dose of 40 Gy in 11 patients and 50.4 Gy in 46 patients. Regarding combination chemotherapy, 31 patients were treated with 5′-DFUR, 19 with capecitabine, and 7 with S1. A total of 31 (54.4 %) patients had >HATE G2 and 7 (12.3 %) had HATE G3. Adjuvant chemotherapy was introduced in 31 patients; 12 involved 5′-DFUR, 7 involved tegafur-uracil/leucovorin, 5 involved capecitabine, 5 involved mFOLFOX6, and 2 involved Cape-OX (data not shown). Recurrence was observed in 19 patients. Recurrence occurred as follows: 3 patients, local region; 7 patients, lung; 4 patients, liver; 2 patients, distant lymph nodes; 1 patient, peritoneal dissemination; 1 patient, bone; and 1 patient, gluteus muscle. One of the 7 patients with HATE G3 had relapse in the liver. The primary local recurrence rate was 5.3 %.

**Table 2:** NACRT regimen, effect of NACRT, and recurrence cases after surgery

NACRT		
Radiation dose	40 Gy	11
	50.4 Gy	46
Combination chemotherapy	5′-DFUR	31
	Capecitabine	19
	S1	7
Histological antitumor effect of NACRT (grade)	1a	8 (14.0 %)
	1b	18 (31.6 %)
	2	24 (42.1 %)
	3	7 (12.3 %)
Recurrence cases	Overall	19 (33.3 %)
	Local	3
	Lung	7
	Liver	4
	Distant lymph nodes	2
	Peritoneum	1
	Bone	1
	Gluteus maximus	1

The results regarding univariate and multivariate analysis of factors associated with postoperative distant organ recurrence are detailed in Table 3. Postoperative distant organ recurrences were more frequent in positive pathological lymph node metastasis (ypN+) patients (*p* = 0.001); the results of multivariate logistic regression analysis indicated that ypN+ was a significant predictor of the incidence of distant organ recurrence (*p* = 0.009). There was no relationship between the incidence of distant organ recurrence and age, gender, surgical procedure, histological depth of tumor, HATE grade, histological type, total radiation dose, and the type of combination chemotherapy.

**Table 3:** Univariate and multivariate analysis of factors associated with postoperative distant organ recurrence

		Number	*p* value of univariate analysis	Odd ratio	*p* value of multivariate analysis	95 % CI
Age	≥ 70	23	0.058	0.525	0.348	0.136–2.090
	< 70	34		Reference		
Gender	Male	42	0.418			
	Female	15				
Surgical procedure	APR + TPE	20	0.812			
	Other operation	37				
Histological depth of tumor	T0-2	19	0.404			
	T3, 4	38				
Histological antitumor effect	G1	26	0.314			
	G2, 3	31				
Histological type	por, muc	5	0.096	0.244	0.201	0.024–2.176
	tub	52		Reference		
Total radiation dose	40 Gy	14	0.524			
	50.4 Gy	43				
Combination chemotherapy	5′DFUR	31	0.442			
	Capecitabine, S1	26				
Pathological lymph nodes metastasis	+	15	0.001	0.156	0.009	0.037–0.608
	–	42		Reference		

**Fig. 1:**
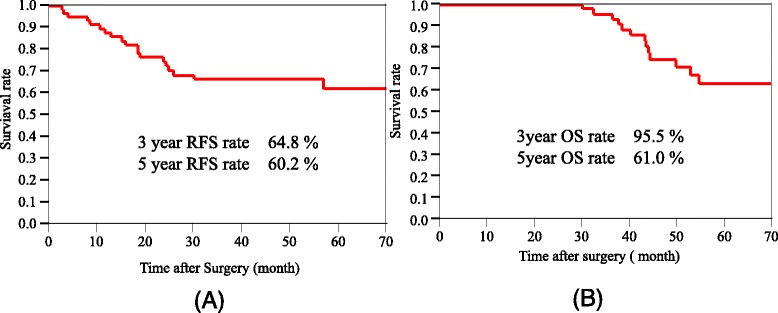
**a** Kaplan–Meier relapse-free survival (median follow-up time 42 months) of all cases. **b** Kaplan–Meier overall survival (median follow-up time 42 months) of all cases. *RFS* relapse-free survival, *OS* overall survival

**Fig. 2:**
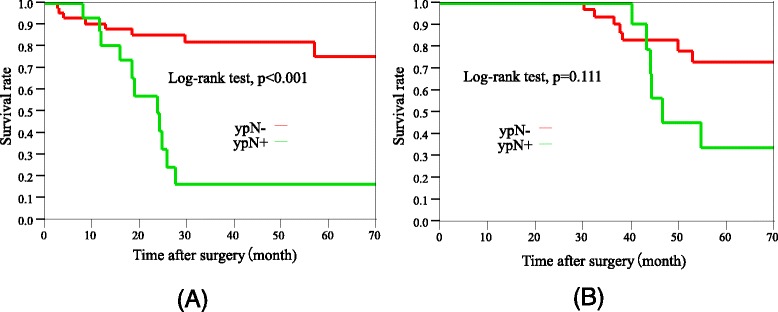
**a** Kaplan–Meier relapse-free survival of ypN+ cases and ypN− cases (median follow-up time 42 months). **b** Kaplan–Meier overall survival of ypN+ cases and ypN− cases (median follow-up time 42 months). *RFS* relapse-free survival, *OS* overall survival

The long-term outcomes are shown in Fig. 1. The median follow-up time regarding RFS and OS was 42 (range 0.5–62.0) months. The 5-year RFS rate was 60.2 % (Fig. 1a), and the 5-year OS rate was 61.0 % (Fig. 1b). In patients with a HATE grade ≥G2, the 3- and 5-year RFS rates were both 71.4 %. There was no significant difference between the RFS rates in the HATE ≥G2 group and the HATE G1 group (3-year RFS rates of 71.4 and 57.7 %, respectively; *p* = 0.256). The 5-year RFS rates in ypStages 0, I, II, IIIa, and IIIB were 85.7, 90.0, 67.3, 26.0, and 0 %, respectively; the 5-year OS rate for these ypStages were 55.6, 100, 69.1, 43.8, and 0 %, respectively. The 5-year RFS rate in the ypN− group was significantly higher than that in the ypN+ group (ypN− vs ypN+, 75.4 vs 16.3 %; *p* < 0.001) (Fig. 2a, b). The 5-year OS rate in the ypN− group was not significantly different from that in the ypN+ group (ypN− vs ypN+, 72.9 vs 33.8 %; *p* = 0.111).

The predictive factors of the effect of NACRT (≥G2) are summarized in Table 4; a rate of neutrophils of <70 % (*p* = 0.002) and a NLR score of <3.0 (*p* = 0.044) in the peripheral blood prior to treatment indicated that NACRT would be significantly more effective. There was no relationship between the efficacy of NACRT and the leukocyte count, the rate of lymphocytes, the hemoglobin and albumin levels, the platelet count, or the CEA score in the peripheral blood.

**Table 4:** Predictive factors in pretreatment peripheral blood regarding the effectiveness of NACRT (≥grade 2)

	Number	Chi-square	*p* value of univariate analysis
Leukocyte count	<8000/mm^3^	0.549	0.459
	≥8000/mm^3^		
Rate of neutrophil	<70 %	9.189	0.002
	≥70 %		
Rate of lymphocyte	≥20 %	2.770	0.096
	<20 %		
NLR score	<3.0	4.070	0.044
	≥3.0		
Hemoglobin level	≥11 g/dl	1.333	0.248
	<11 g/dl		
Albumin level	≥3.5 g/ml	0.395	0.530
	<3.5 g/ml		
Platelet count	<300,000/μl	0.713	0.398
	≥300,000/μl		
CEA score	<10 ng/ml	0.021	0.886
	≥10 ng/ml		

Yates chi-square test

*NLR* neutrophil-to-lymphocyte ratio, *CEA* carcinoembryonic antigen

## Discussion

Treatment with NACRT in patients with LARC has been beneficial in terms of local tumor control and sphincter preservation [11, 12]. However, the most important goal of rectal cancer therapy is improvement in survival. In the present study, the percentage of patients who achieved a HATE grade ≥G2 was 54.4 and 12.3 % achieved HATE grade G3 (pCR). Although the efficacy of NACRT regarding local tumor control was proven, RFS and OS were not significantly prolonged by NACRT. The 5-year OS rate in patients with ypStage II disease in our study was 69.1 %. This outcome was similar to that of Japanese LARC patients with pStage IIIa disease. Furthermore, the 5-year OS rate (43.8 %) of patients with ypStage IIIa in our study was similar to that of Japanese LARC patients with pStage IIIb disease [10]. This observation demonstrated that NACRT was effective for the downstaging of LARC but did not contribute to prolongation of the OS of LARC patients. Similar to our outcome, no survival-prolonging effects regarding NACRT were demonstrated in previous reports [13, 14].

Schrag et al. reported that >25 % of patients with LARC had distant organ metastasis [15]. We also found that 16 patients (28.1 %) exhibited recurrence in distant organs. Regarding multivariate analysis of the factors associated with postoperative distant organ recurrence, ypN+ was found to be a high-risk factor, where the RFS rate of ypN+ patients was significantly lower than that of ypN− patients. Fokas et al. reported that a higher ypN category after preoperative CRT was the strongest prognostic factor regarding multivariate analysis [16]. There have been some other studies that have reported on other predictive factors related to recurrence after NACRT. Toiyama et al. found that C-reactive protein was a promising predictor of recurrence and prognosis in patients with rectal cancer after NACRT [17]. Further, factors such as fibroblast growth factor receptor 2 overexpression [18], ALDH1 [19], and upregulated polo-like kinase in rectal cancer [20] were reported to be predictors of recurrence after NACRT. Further study will be necessary to clarify the predictive factors regarding NACRT and to facilitate improvement of long-term outcomes of NACRT.

Many predictive biomarkers concerning the antitumor effect of NACRT have been reported. Patients who responded well to chemoradiothetapy (CRT) for rectal cancer had a significantly higher number of pre-CRT lymphocytes [21]; sustaining a high blood lymphocyte count during NACRT was predictive of achieving a pCR in rectal cancer [22]. In addition to this, it has been reported that predictive biomarkers for NACRT were tumor differentiation grade and B cell lymphoma 2 expression [23]. As a negative predictive factor, Flanagan et al. reported that the increased X-linked inhibitor of apoptosis protein may be a useful indicator of NACRT resistance in rectal cancer tissues [24]. In our study, positive predictive factors regarding the effectiveness of NACRT (≥G2) were a neutrophil rate <70 % and an NLR score <3.0, indicating that the therapeutic efficacy of NACRT may be lower if inflammation is present. As similar to our data, Krauthamer et al. reported that an NLR <5 and a serum albumin level >3.5 mg/dL may be positively related to complete pathological response after NACRT in patients with clinical stage III LARC [25]. For improving the prognosis of LARC patients, it is necessary to select other positive biomarkers for predicting the effectiveness of NACRT by analyzing pretreatment rectal cancer specimens or blood.

In the present study, we administered oral fluoropyrimidine anticancer agents, such as 5′-DFUR, capecitabine, and S1 with radiotherapy and the pCR rate was 12.3 %. Similar results were obtained using neoadjuvant single-agent capecitabine plus radiotherapy for LARC [26]. Recently, cytotoxic agents such as irinotecan and oxaliplatin plus fluorouracil have been administrated for combination chemotherapy regarding NACRT [27, 28]. Wong et al. reported that the efficacy of capecitabine plus irinotecan with radiotherapy and the efficacy of capecitabine plus oxaliplatin with radiotherapy were similar in a neoadjuvant setting for LARC patients. However, it remains uncertain as to whether or not the addition of a second cytotoxic agent enhances the effectiveness of fluorouracil plus radiotherapy [27]. The ACCORD 12 trial investigated the value of two different NACRT regimens involving CAP45 (45 Gy radiotherapy plus capecitabine) or CAPOX50 (50 Gy radiotherapy plus capecitabine plus oxaliplatin). Unfortunately, there were no significant differences in clinical results such as OS and disease-free survival (DFS) at 3 years, where the authors did not recommend the administration of oxaliplatin with radiotherapy [28]. To evaluate the best combination chemotherapy with radiotherapy, it will be essential to plan further large-scale randomized clinical studies.

In our study, adjuvant chemotherapy was introduced in only 31 (54.4 %) patients. The low introduction rate of adjuvant chemotherapy possibly resulted in no improvement in survival time. There have been several studies that have reported no benefit of adjuvant chemotherapy for LARC after NACRT. Sainato et al. reported that the addition of adjuvant chemotherapy (5-fluorouracil-folinic acid) in patients with LARC treated with NACRT did not improve 5-year OS and DFS and had no impact on the rate of distant metastasis [29]. Rodel et al. reported that LARC patients after NACRT had a low compliance to adjuvant chemotherapy [30]. Additionally, the ACT regimen (adriamycin, cytoxan, and taxol) had no effect in LARC patients after NACRT [31, 32]. In contrast, Hong et al. reported that adjuvant FOLFOX (oxaliplatin, 5-fluorouracil, and leucovorin) improved DFS relative to fluorouracil plus leucovorin in patients with LARC after NACRT [33], but prolongation of OS was not demonstrated. It seems likely that improvement in prognosis, especially prolongation of OS, is difficult after the introduction of ACT after NACRT.

Recently, neoadjuvant chemotherapy (NAC) has been administered to LARC patients instead of NACRT. Schrag et al. [15] reported that the outcomes of LARC patients who underwent FOLFOX chemotherapy without radiotherapy in a neoadjuvant setting were not inferior to those of NACRT. In this study, all patients exhibited tumor regression, and TME and the pCR rate after chemotherapy alone were 8 of 32; the 4-year local recurrence and DFS rates were 0 and 84 %, respectively [15]. Xiao et al. reported that although there were no pCR cases, sandwich-like NAC with bevacizumab was safe and effective for LARC [34]. To confirm the results of NAC, a randomized trial (PROSPECT trial) has been initiated in North America [35]. In the near future, NAC may become the main therapy for LARC after NACRT.

Our study does lack in new insights, however, the clinical outcomes for Japanese LARC patients after NACRT have not been established yet. In the future, a multicenter prospective randomized controlled study in Japan is necessary to validate the usefulness, efficacy, and prognosis of NACRT in Japanese LARC patients.

## Conclusions

In the present study, NACRT for Japanese LARC patients was found to be an effective treatment in reducing local recurrence but did not suppress distant organ recurrence. NACRT could not prolong RFS and OS. It will be necessary to change the NACRT regimen and to further investigate preoperative multimodal therapy for LARC such as NAC.

### Consent

Written informed consent was obtained from the locally advanced rectal carcinoma patients for induction of NACRT.
